# Population-Based Estimates of Hepatitis E Virus–Associated Mortality in Bangladesh

**DOI:** 10.1093/infdis/jiaf134

**Published:** 2025-03-13

**Authors:** Repon C Paul, Heather F Gidding, Arifa Nazneen, Kajal C Banik, Shariful Amin Sumon, Kishor K Paul, Arifa Akram, M Salim Uzzaman, Alexandra Tejada-Strop, Saleem Kamili, Stephen P Luby, Andrew Hayen, Emily S Gurley

**Affiliations:** International Centre for Diarrhoeal Disease Research, Bangladesh, Dhaka, Bangladesh; National Perinatal Epidemiology and Statistics Unit, Centre for Big Data Research in Health, Faculty of Medicine and Health, University of New South Wales, Australia; School of Population Health, Faculty of Medicine and Health, University of New South Wales, Australia; Health and Clinical Analytics Lab, Sydney School of Public Health, Faculty of Medicine and Health, University of Sydney, Australia; International Centre for Diarrhoeal Disease Research, Bangladesh, Dhaka, Bangladesh; International Centre for Diarrhoeal Disease Research, Bangladesh, Dhaka, Bangladesh; International Centre for Diarrhoeal Disease Research, Bangladesh, Dhaka, Bangladesh; International Centre for Diarrhoeal Disease Research, Bangladesh, Dhaka, Bangladesh; School of Population Health, Faculty of Medicine and Health, University of New South Wales, Australia; Institute of Epidemiology, Disease Control and Research, Dhaka, Bangladesh; Institute of Epidemiology, Disease Control and Research, Dhaka, Bangladesh; Division of Viral Hepatitis, Centers for Disease Control and Prevention, Atlanta, Georgia; Division of Viral Hepatitis, Centers for Disease Control and Prevention, Atlanta, Georgia; Infectious Diseases and Geographic Medicine, Stanford University, California; School of Public Health, University of Technology Sydney, Australia; International Centre for Diarrhoeal Disease Research, Bangladesh, Dhaka, Bangladesh; Department of Epidemiology, Johns Hopkins Bloomberg School of Public Health, Baltimore, Maryland

**Keywords:** burden, endemic countries, hepatitis E, mortality, population-based estimates

## Abstract

**Background:**

Hepatitis E virus (HEV) is endemic in many resource-poor countries. Despite an available vaccine, data on HEV-associated mortality are scarce, hindering informed decisions. This study aims to estimate the population-based rate of HEV-specific mortality in Bangladesh.

**Methods:**

During December 2014 to September 2017, we conducted surveillance in 6 tertiary hospitals in Bangladesh. Patients aged ≥14 years with acute jaundice were recruited, tested for IgM anti-HEV, and followed up postdischarge. A mortality survey in the hospital catchment areas identified deaths associated with acute jaundice, including maternal deaths, stillbirths, and neonatal deaths delivered by a mother with acute jaundice during pregnancy, as confirmed by 2 independent physicians reviewing verbal autopsy data.

**Results:**

Out of 1925 patients with acute jaundice enrolled in the surveillance hospitals, 302 died, with 28 (9%) testing positive for IgM anti-HEV. In the hospital catchment areas, the team identified 587 jaundice-associated deaths, including 25 maternal deaths. Combining hospital-based surveillance and mortality survey data, the study estimated 986 (95% CI, 599–1338) HEV-associated deaths annually among individuals aged ≥14 years in Bangladesh, including 163 (95% CI, 57–395) maternal deaths. Additionally, 279 (95% CI, 101–664) stillbirths and 780 (95% CI, 365–1297) neonatal deaths were attributed to HEV infection annually.

**Conclusions:**

Prior Global Burden of Disease studies presented wildly varying modeling estimates of HEV-associated annual deaths, ranging from 50 000 in 2013 to 1932 in 2019. This study is the first to directly measure population-based estimates of mortality in Bangladesh, which can be used to determine the cost-effectiveness of hepatitis E vaccination and other interventions.

## Teaser

This study provides the first population-based estimate of hepatitis E virus–associated mortality in Bangladesh, revealing higher mortality than global estimates. Findings suggest underreporting of hepatitis E virus deaths and inform the cost-effectiveness of vaccination and other public health interventions.

Hepatitis E virus (HEV) causes acute liver infection: transmission of genotypes 1 and 2 occurs among humans through the fecal-oral route, while genotypes 3 and 4 are zoonotic and primarily infect humans through the consumption of undercooked meat [1, 2]. In low- and middle-income countries (LMICs), where fecal contamination of drinking water is common, genotypes 1 and 2 pose significant public health concerns [1, 2]. HEV disproportionately affects vulnerable populations, particularly pregnant women, leading to maternal mortality and adverse outcomes such as stillbirth and neonatal death [1, 3, 4]. Despite this, HEV remains underprioritized in global health agendas, partly due to a lack of reliable data on its disease burden [5]. In LMICs, where HEV infection is a leading cause of acute viral hepatitis [1], population-based studies estimating the disease burden are scarce due to inadequate surveillance systems and limited vital registration of cause-specific deaths.

Existing estimates of the burden of HEV infection rely on mathematical models, including the Global Burden of Disease (GBD) effort, and were built on several strong assumptions [6, 7]. These assumptions include predicting the proportion of people with antibodies against HEV who develop clinical disease, comparing HEV-associated mortality between HEV-endemic LMICs and high-income countries with effective vital registration, and extrapolating the likelihood of death among hospitalized patients with hepatitis E to nonhospitalized patients with severe cases. Notably, the global annual HEV-related death toll projections by these models have varied significantly, from 70 000 in 2005 to 1932 in 2019 [6–10].

HEV infection is preventable through sanitation, adherence to safe food and water precautions, and potentially vaccination. Two hepatitis E vaccine candidates, following nonhuman primate experiments, progressed to clinical trials in humans [11, 12]. One of these vaccines, HEV 239, was licensed in China in 2011 and Pakistan in 2020 for use among residents aged ≥16 years [13]. A major barrier to recommending the vaccine in routine national vaccination programs in other countries, however, is the lack of confidence in the wide-ranging burden of disease estimates and a dearth of population-based measurements of HEV-associated mortality [14].

From 2014 to 2017, the International Centre for Diarrhoeal Disease Research, Bangladesh, in collaboration with the Institute of Epidemiology, Disease Research and Control of the Government of Bangladesh and the US Centers for Disease Control and Prevention, conducted a hospital-based surveillance study in Bangladesh to identify patients admitted with acute jaundice [15]. Yet, estimating morality burden from hospital-based studies may not fully represent the general population due to health care access disparities in many LMICs [16]. We conducted a mortality survey in the catchment areas of surveillance hospitals and calculated population-based estimates of HEV-specific mortality by combining data from the hospital-based surveillance and mortality survey.

## METHODS

### Hospital-Based Acute Jaundice Surveillance

**Figure 1.**
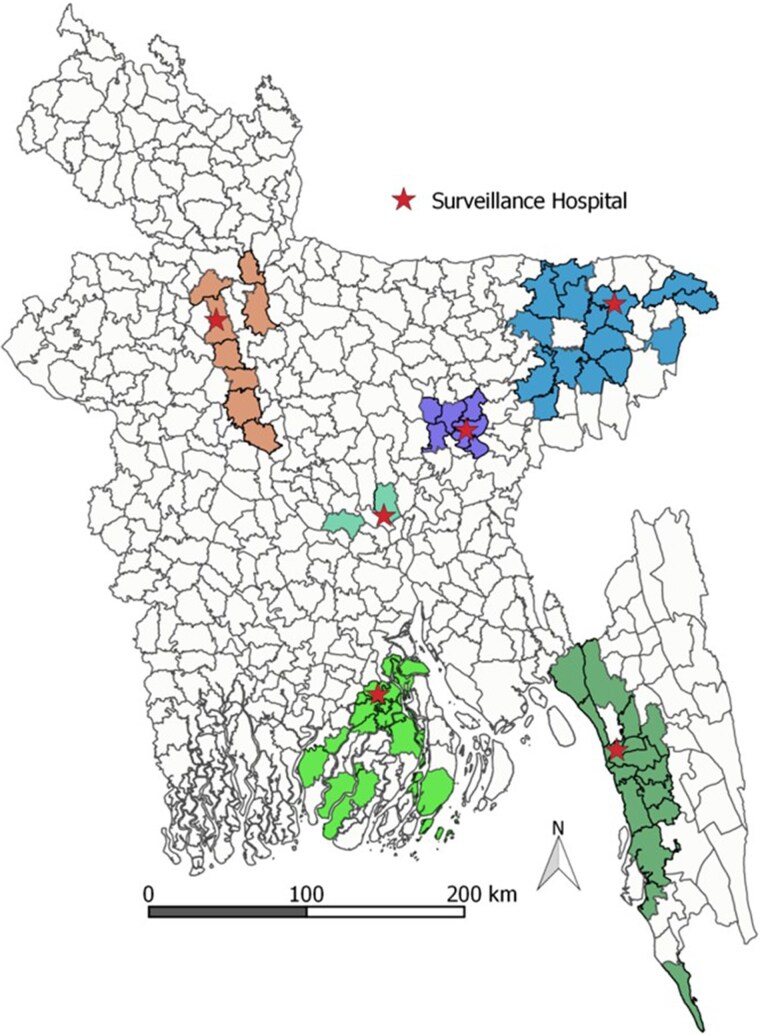
Map of Bangladesh showing the acute jaundice surveillance sites and the selected catchment areas (subdistricts) for mortality survey, December 2014 to September 2017. Surveillance hospitals, red stars; catchment areas, colored regions.

Hospital-based acute jaundice surveillance details are available elsewhere [15]. Briefly, between December 2014 and September 2017, a study investigated acute jaundice in 6 randomly selected tertiary hospitals across 5 divisions in Bangladesh (Figure 1). These hospitals were chosen to ensure geographic representation within the country, which has a land area <57 000 square miles—comparable to a midsized US state. Patients aged ≥14 years were recruited if they exhibited acute jaundice, defined as new onset of yellow sclera or skin during the past 3 months that persisted on admission day. Blood samples from all recruited patients were tested for IgM anti-HEV by an enzyme-linked immunosorbent assay with a sensitivity of 98% to 100% and a specificity of 95% to 100% (Beijing Wantai Biologic Pharmacy Enterprise Co, Ltd). Patients positive for IgM anti-HEV antibodies were considered to have acute HEV infection.

Enrolled patients were followed during their hospitalization and for 3 months postdischarge to ascertain vital status and pregnancy outcomes for pregnant women. However, postdischarge follow-up was not possible for patients admitted after June 2017, as surveillance ended on 30 September 2017.

### Mortality Survey in Hospital Catchment Areas

#### Selection of Hospital Catchment Areas

**Figure 2.**
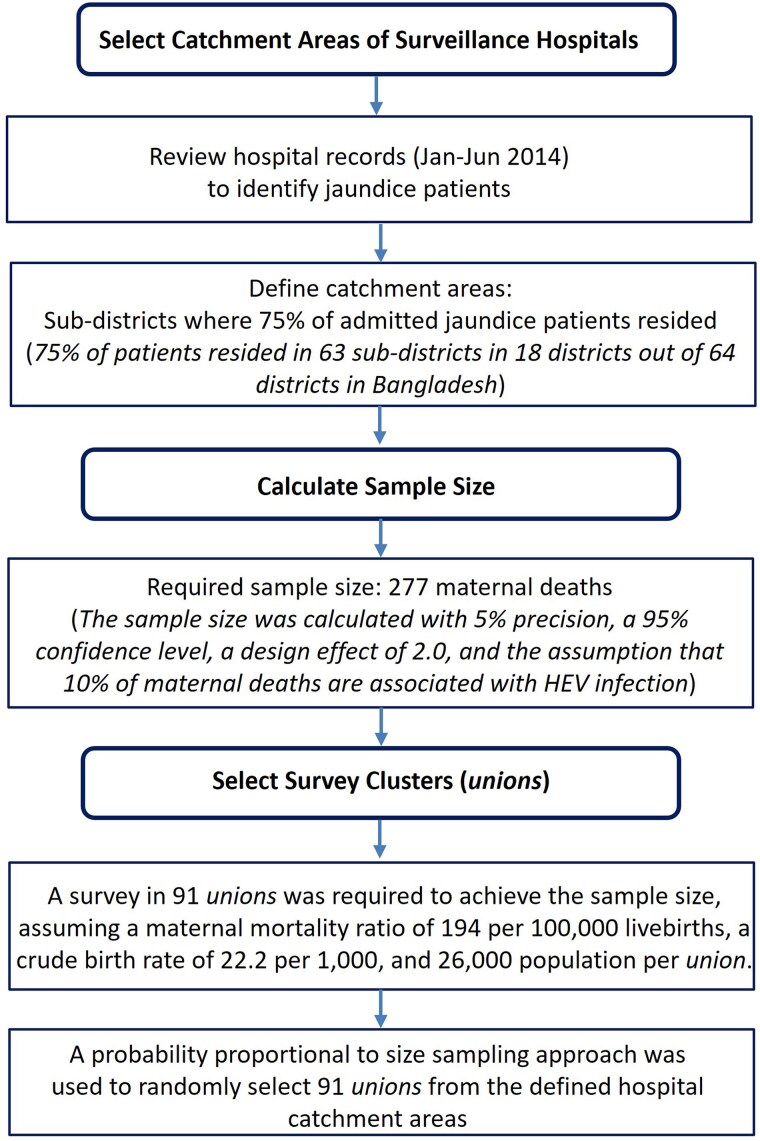
Flowchart illustrating the process of selecting catchment areas of surveillance hospitals and clusters for the mortality survey in Bangladesh. Abbreviation: HEV, hepatitis E virus.

The study team reviewed the hospital records of patients with jaundice who were admitted from January to June 2014 to identify the primary catchment areas for each surveillance hospital. Catchment areas were defined as the subdistricts where 75% of the admitted patients resided (Figure 2). By applying this criterion, 75% of patients with jaundice who were admitted to the surveillance hospitals were from 63 subdistricts across 18 of 64 districts in Bangladesh. Subdistricts in Bangladesh consist of a median 15 unions (IQR, 7–20), the smallest administrative units, each with an average population of 26 000 people [17].

#### Selection of Survey Clusters

**Figure 3.**
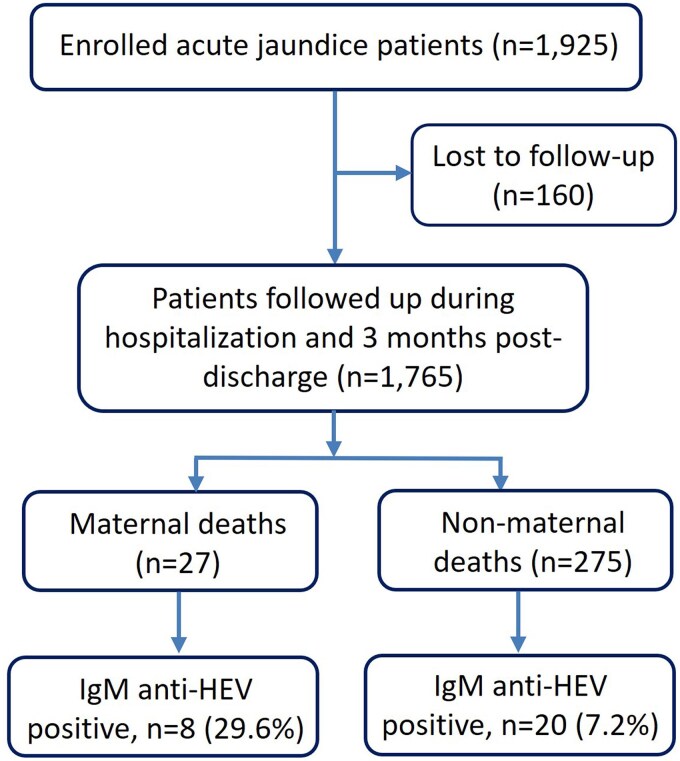
Flowchart of patients with acute jaundice enrolled in the surveillance hospitals from December 2014 to September 2017. Abbreviation: HEV, hepatitis E virus.

Estimating rare events, such as maternal deaths, requires a large sample size [18]. Since our study aimed to estimate maternal mortality associated with HEV infection with acceptable precision, we assumed that it would also provide sufficient power for estimating adult mortality, neonatal deaths, and stillbirths associated with HEV infection. The sample size for estimating maternal mortality was determined by the formula for estimating proportions [19], assuming that 10% of maternal deaths are HEV related [20]. With 5% precision, a 95% confidence level, and a design effect of 2.0, we calculated a required sample size of 277 maternal deaths in the surveillance hospitals’ catchment areas. To achieve this size, the survey was conducted in 91 unions, collecting mortality data from the preceding 3 years (Figure 3). This calculation was based on an assumed maternal mortality ratio of 194 per 100 000 live births [21] and a crude live birth rate of 22.2 per 1000 population [22]. A “probability proportional to size sampling” approach was used to randomly select 91 unions from the defined hospital catchment areas [23].

#### Mortality Survey

Between November 2014 and February 2017, a mortality survey was undertaken across 91 unions to identify jaundice-associated deaths. The survey also identified stillborn babies and liveborn babies who died during the neonatal period when delivered by mothers with jaundice during pregnancy, as well as all-cause maternal deaths. Jaundice was defined as having new onset of yellow sclera or skin during the illness prior to death. Neonatal deaths were those occurring within the first 28 days of life, while stillbirths were defined as babies delivered after 28 weeks of gestation with no signs of life [24]. Maternal death was defined as the death of a woman while pregnant or within 42 days of pregnancy termination, excluding unintentional injuries or unrelated incidental causes [24].

In urban communities, we conducted a house-to-house survey, whereas in rural communities, we used a low-cost community knowledge approach, as detailed elsewhere [25]. Out of 91 unions, 25 (27%) were urban, closely matching the urban population percentage (23%) in the 2011 Bangladesh Population Census [26]. For jaundice-associated deaths and all-cause maternal deaths, a standardized World Health Organization verbal autopsy questionnaire was administered to collect detailed information on signs, symptoms, and duration of illness preceding death to determine the underlying and immediate cause of death [27]. Two physicians independently assessed verbal autopsy questionnaires, assigning *ICD-10* cause-of-death codes. Discordant cases prompted discussion for consensus, with a third physician intervening if disagreement persisted.

### Data Analysis

We calculated the HEV-associated mortality rate for individuals aged ≥14 years, maternal mortality ratio, and stillbirth and neonatal mortality rate for all catchment areas combined. The population in 2014 served as our midyear reference—considering that our data collection spanned November 2014 to February 2017, with about 60% collected in 2015—and we collected mortality data for the prior 3 years. For rural unions, the 2014 population was projected per the 2011 Bangladesh census and an annual growth rate of 1.37% (Supplementary Appendix 1) [28]. In urban unions, we counted the household population at the time of the mortality survey. We projected the population aged ≥14 years, assuming that 67.7% of total population in 2014 was aged ≥14 years [28]. The number of live births in 2014 was estimated by assuming a crude birth rate of 22.2 per 1000 population per the 2014 Bangladesh Demographic and Health Survey [22].

The mortality survey collected information on chronic and acute jaundice-associated deaths; yet, for incidence calculation, only deaths associated with acute jaundice were considered, defined as new onset of yellow eyes or skin within the 3 months prior to death. Because we used the same case definition of acute jaundice in hospitals and hospital catchment areas, we assumed that the illness in people who died in the catchment areas was similar to that of people who died in the study hospitals. To calculate HEV-associated mortality in the hospital catchment areas, we multiplied the estimate for jaundice-associated mortality in the hospital catchment areas by the proportion of HEV infections among acute jaundice deaths in the surveillance hospitals.

Recognizing HEV's additional risk during pregnancy [1, 2] and its varying age-specific infection rates and severity [29], we separately projected HEV-associated deaths among pregnant women and nonpregnant persons aged ≥14 years (14–44, 45–59, and ≥60 years). The projected deaths in both groups were aggregated to estimate the total number of HEV-associated deaths in the hospital catchment areas. We used the following equation to estimate mortality associated with HEV in the hospital catchment areas for the population aged ≥14 years:


$$\frac{D_{m\;}\frac{E_{m}}{J_{m}}+\sum \;D_{i}\;\frac{E_{i}}{J_{i}}}{P\;*\;3}\times 100,000$$

P= Estimated population aged ≥14 years in the study areas in 2014

$D_{m}=$ Number of maternal deaths associated with acute jaundice in the 3-year period in the study areas

$J_{m}=$ Number of maternal deaths associated with acute jaundice in the surveillance hospitals

$E_{m}=$ Number of laboratory-confirmed HEV cases among maternal deaths associated with acute jaundice in the surveillance hospitals

$D_{i}=$ Number of age-specific nonmaternal deaths associated with acute jaundice in the 3-year period in the study areas

$J_{i}=$ Number of age-specific nonmaternal deaths associated with acute jaundice in the surveillance hospitals

$E_{i}=$ Number of age-specific laboratory-confirmed HEV cases among nonmaternal deaths associated with acute jaundice in the surveillance hospitals

Similarly, the following formulas were used to calculate HEV-associated maternal mortality, neonatal mortality, and stillbirth:


$$\mathrm{Maternal}\;\mathrm{mortality}\;\mathrm{ratio}\;\mathrm{associated}\;\mathrm{with}\;\mathrm{HEV}=\left(\frac{\frac{D_{m}}{3}}{B}\right)\left(\frac{E_{m}}{J_{m}}\right)\times 100,000$$

$$\mathrm{Stillbirth}\;\mathrm{rate}\;\mathrm{associated}\;\mathrm{with}\;\mathrm{HEV}=\left(\frac{\frac{S}{3}}{B}\right)\left(\frac{S_{e}}{S_{j}}\right)\times 100,000$$

$$\mathrm{Neonatal}\;\mathrm{mortality}\;\mathrm{rate}\;\mathrm{associated}\;\mathrm{with}\;\mathrm{HEV}=\left(\frac{\frac{N}{3}}{B}\right)\left(\frac{N_{e}}{N_{j}}\right)\times 100,000$$

$\;D_{m},\;\;J_{m}\;$ , and $E_{m}=$ as previously defined

B= Estimated live births in the study areas in 2014

S= Number of stillbirths delivered by a mother with acute jaundice during pregnancy in the 3-year period in the study areas

$S_{j}=$ Number of stillbirths delivered by a mother with acute jaundice during pregnancy in the surveillance hospitals

$S_{e}=$ Number of stillbirths delivered by a mother with acute HEV during pregnancy in the surveillance hospitals

N= Number of neonatal deaths born to a mother with acute jaundice during pregnancy in the 3-year period in the study areas

$N_{j}=$ Number of neonatal deaths born to a mother with acute jaundice during pregnancy in the surveillance hospitals

$N_{e}=$ Number of neonatal deaths born to a mother with acute HEV during pregnancy in the surveillance hospitals

We used nonparametric bootstrapping [30] to estimate 95% CIs for HEV-associated mortality for the population aged ≥14 years. For maternal mortality, stillbirth, and neonatal mortality associated with HEV infection, 95% CIs were calculated by multiplying the lower and upper bounds of jaundice-associated mortality in the hospital catchment areas by the respective lower and upper bounds of HEV infection rates among acute jaundice deaths in the study hospitals. The 95% CI of the proportion of HEV among acute jaundice deaths in the study hospitals was calculated via the Wilson method for a binomial distribution [31], and the 95% CI for jaundice-associated deaths in the hospital catchment areas was calculated with the 95% CI for the mean of a Poisson distribution. We performed a sensitivity analysis to check how the estimates of HEV-associated mortality vary when restricted to 2- and 1-year recall periods.

### Population Mortality Estimates

The HEV-associated mortality estimates generated from the study hospitals and mortality surveys were considered representative of the hospital catchment areas, and with surveillance hospitals covering 5 of 7 administrative divisions, we expected that results could be extrapolated to the whole country (Figure 1). The rural to urban population ratio of our study matched the national ratio, which was important because urbanicity is a known risk factor for HEV infection [26]. We therefore used our morality estimates to extrapolate the total number of HEV-associated deaths among the population aged ≥14 years, maternal deaths associated with HEV, and stillborn babies and liveborn babies who died during the neonatal period when delivered by mothers with acute HEV infection during pregnancy in 2014 in Bangladesh.

### Ethical Approval and Consent

The study protocol was reviewed and approved by the institutional review board of the International Centre for Diarrhoeal Disease Research, Bangladesh (protocol PR-14060). In the surveillance hospitals, written informed consent was obtained from patients aged >17 years. For patients aged 14 to 17 years, assent was taken from the patients alongside parental consent. Written consent was sought from guardians or accompanying attendants if patients were unable to provide consent themselves because of illness. In the mortality survey, written consent was obtained from a family member directly involved in caregiving during the deceased's illness period.

## RESULTS

### Deaths Among Hospitalized Patients With Acute Jaundice

In the 6 study hospitals, 1925 patients with acute jaundice were identified during December 2014 to September 2017; all consented for enrollment. Of them, 1765 (92%; including 173 pregnant women) were followed up during hospitalization and 3 months postdischarge. Among these patients, 302 died (17%), including 27 maternal deaths and 28 (9%; 95% CI, 6%–13%) testing positive for IgM anti-HEV. Among the maternal deaths, 8 (30%; 95% CI, 16%–49%) were positive for IgM anti-HEV (Table 1).

**Table 1.** Hepatitis E Cases Among Patients With Acute Jaundice Who Died During Hospitalization or Within 3 Months of Hospital Discharge in 6 Tertiary Hospitals in Bangladesh, December 2014–September 2017

Characteristic	Patients With Acute Jaundice Who Died^a^ (n = 302)	IgM Anti-HEV Positive (n = 28)	
		No.	% (95% CI)
Death			
Maternal	27	8	29.6 (15.8–48.5)
Nonmaternal	275	20	7.2 (4.8–11.0)
Sex			
Male	178	13	7.3 (4.3–12.1)
Female	97	7	7.2 (3.5–14.2)
Age group, y			
14–44	96	14	14.6 (8.9–23.6)
45–59	86	3	3.5 (1.2–9.8)
≥60	93	3	3.2 (1.1–9.1)

Abbreviation: HEV, hepatitis E virus.

^a^Defined as new onset of either yellow eyes or skin during the past 3 months, continuing on the day of admission.

Among the 173 pregnant women followed, 125 (72%) had live births, 27 (16%) experienced stillbirths, 11 (6%) had miscarriages, and 10 (6%) died before the pregnancy outcome. Of the enrolled pregnant women, 66 (38%) tested positive for IgM anti-HEV. Of the 27 stillbirths, 6 (22%; 95% CI, 11%–41%) were delivered by mothers with IgM anti-HEV positivity. Among 125 live births, 15 (12%) died within 28 days of birth (neonatal deaths), with 10 (67%; 95% CI, 42%–85%) born to mothers who were HEV positive.

### Acute Jaundice Deaths in the Hospital Catchment Areas

In the catchment areas of study hospitals, the team identified 33 794 deaths among persons aged ≥14 years who died in the 3 years preceding survey (Table 2). Among these, 277 (0.8%) were maternal deaths. Of the maternal deaths, 25 (9%) involved acute jaundice, as opposed to 587 (1.7%) among the nonmaternal deaths. There were 57 stillbirths and 53 neonatal deaths delivered by mothers with acute jaundice during pregnancy.

**Table 2.** Deaths Associated With Acute Jaundice in the Catchment Areas of the 6 Surveillance Hospitals, Bangladesh, During the 3-Year Period Prior to the Survey

	Deaths Aged ≥14 y, No. (%; 95% CI)		
Characteristic	Maternal	Nonmaternal	Total
Deaths identified in the study areas^a^	277	33 517	33 794
Deaths with jaundice^b^ prior to death	28 (10.1; 7.0–14.2)	778 (2.3; 2.2–2.5)	806 (2.4; 2.2–2.6)
Deaths with acute jaundice^c^ prior to death	25 (9.0; 6.1–12.9)	587 (1.7; 1.6–1.9)	612 (1.8; 1.7–1.9)

Survey period: November 2014–February 2017.

^a^There were 2 more maternal deaths for whom their caregivers refused to participate in this study.

^b^Defined as having yellow eyes or skin prior to death.

^c^Defined as new onset of either yellow eyes or skin during the 3 months prior to death.

### Demographic Characteristics of Acute Jaundice Deaths

#### Surveillance Hospitals

Among nonmaternal acute jaundice deaths, two-thirds were male and one-third were aged ≥60 years (Table 3). Eighty-two percent resided in rural areas, and 40% had no formal education. In most cases (72%), jaundice duration before hospitalization was <1 month, and over three-quarters of the deaths occurred at home postdischarge.

**Table 3.** Demographic Characteristics of Patients With Acute Jaundice in the Study Hospitals Who Died During Hospitalization or Within 3 Months of Discharge and Deaths Associated With Acute Jaundice in the Hospital Catchment Areas in the 3 Years Presurvey

	Deaths in Hospitals, No. (%)^a^			Deaths in Hospital Catchment Areas, No. (%)^a^			
Characteristic	Maternal (n = 27)	Nonmaternal (n = 275)	Total (n = 302)	Maternal (n = 25)	Nonmaternal (n = 587)	Total (n = 612)	*P* Value^b^
Sex							.288
Male	…	178 (65)	178 (59)	…	383 (65)	383 (63)	
Female	27 (100)	97 (35)	124 (41)	25 (100)	204 (35)	229 (37)	
Age, y							<.001
14–44	27 (100)	96 (35)	123 (41)	25 (100)	158 (27)	183 (30)	
45–59	0	86 (31)	86 (29)	0	160 (27)	160 (26)	
≥60	0	93 (34)	93 (31)	0	269 (46)	269 (44)	
Area of residence							.036
Rural	17 (63)	232 (84)	249 (82)	19 (76)	517 (88)	536 (88)	
Urban	10 (37)	43 (16)	53 (18)	6 (24)	70 (12)	76 (12)	
Education							<.001
None	4 (15)	118 (43)	122 (40)	5 (20)	306 (52)	311 (51)	
Class 1–4	8 (30)	72 (26)	80 (27)	3 (12)	61 (10)	64 (11)	
Class 5–9	9 (33)	55 (20)	64 (21)	16 (64)	160 (27)	176 (29)	
Class ≥10	6 (22)	30 (11)	36 (12)	1 (4)	49 (8)	50 (8)	
Unknown	0	0	0	0	11 (2)	11 (2)	
Duration of jaundice at time of death, mo							.002
≤1	21 (78)	197 (72)	218 (72)	16 (64)	356 (61)	372 (61)	
>1 to ≤2	5 (19)	49 (18)	54 (18)	1 (4)	138 (24)	139 (23)	
>2 and ≤3	1 (4)	29 (11)	30 (10)	8 (32)	93 (16)	101 (17)	
Sought care from a qualified health care provider^c^							
First visit	…	…	…	15 (60)	343	358 (59)	
First or second visit	…	…	…	20 (80)	481	501 (82)	
Any visit	…	…	…	21 (84)	544	565 (92)	
Place of death							.103
Health facility	14 (52)	51 (19)	65 (22)	15 (60)	85	100 (16)	
Home	13 (48)	224 (81)	237 (78)	7 (28)	480	487 (80)	
In transit	…	…	…	3 (12)	22	25 (4)	

^a^Among persons aged ≥14 years.

^b^χ^2^ test comparing total deaths in hospitals and hospital catchment areas by patient characteristics.

^c^Health facility, qualified medical doctor, paramedic, nurse.

#### Hospital Catchment Areas

The sex ratio of acute jaundice deaths in the hospital catchment areas was similar to that in the surveillance hospitals (*P* = .288; Table 3). However, those who died in the catchment areas were significantly older than those who died in the hospital surveillance study (*P* < .001).

### Population-Based Estimate of HEV-Associated Mortality

In the 91 unions selected from the catchment areas of 6 study hospitals, the projected population aged ≥14 years was approximately 1.6 million, with an estimated 52 326 live births in 2014 (Supplementary Appendix 1). We estimated HEV-associated mortality as 0.9 (95% CI, .6–1.3) per 100 000 population aged ≥14 years (Table 4). The maternal mortality ratio associated with HEV was estimated as 4.7 (95% CI, 1.6–11.4) per 100 000 live births. We estimated the HEV-associated stillbirth rate as 8.1 (95% CI, 2.9–19.2) and neonatal mortality rate as 22.5 (95% CI, 10.5–34.5) per 100 000 live births. We extrapolated these estimates to the whole population of Bangladesh and estimated that in 2014 there were 986 (95% CI, 599–1338) HEV-associated deaths among the population aged ≥14 years, including 163 (95% CI, 57–395) maternal deaths. Additionally, we estimated 279 (95% CI, 101–664) stillbirths and 780 (95% CI, 365–1297) neonatal deaths associated with HEV infection in 2014 in Bangladesh.

**Table 4.** Estimation of HEV-Associated Mortality in the Catchment Areas of 6 Acute Jaundice Surveillance Hospitals in Bangladesh, 2014

Mortality Estimation (Indicators)	No. (95% CI)
Estimated population and live births in the hospital catchment areas^a^	
Population aged ≥14 y: *P*	1 595 697
Live births: *B*	52 326
Surveillance hospitals	
Maternal deaths	…
Maternal deaths with acute jaundice: $J_{m}$	27
HEV cases among maternal deaths with acute jaundice: $E_{m}$	8 (4–13)
Nonmaternal deaths	
Nonmaternal deaths with acute jaundice: *J*	275
HEV cases among nonmaternal deaths with acute jaundice: ∑$E_{i}$	20 (13–30)
Stillbirths	
Stillbirths delivered by mothers with acute jaundice: $S_{j}$	27
Stillbirths delivered by mothers who were HEV positive: $S_{e}$	6 (3–11)
Neonatal deaths	
Neonatal deaths born to mothers with acute jaundice: $N_{j}$	15
Neonatal death cases born to mothers who were HEV positive: $N_{e}$	10 (6–13)
Hospital catchment areas	
Maternal deaths with acute jaundice: $D_{m}$	25 (16–37)
Nonmaternal deaths with acute jaundice: D	587 (401–772)
Expected number of nonmaternal HEV deaths^b^: $\sum_{i}^{D}\;\;\frac{E_{i}}{J_{i}}$	37
Stillbirths delivered by mothers with acute jaundice: *S*	57 (43–74)
Neonatal deaths delivered by mothers with acute jaundice: *N*	53 (40–69)
HEV-associated mortality	
HEV-associated mortality per 100 000 population aged ≥14 y	0.93 (.6–1.3)
Maternal mortality ratio due to HEV per 100 000 live births	4.7 (1.6–11.4)
Stillbirth rate due to HEV per 100 000 live births	8.1 (2.9–19.2)
Neonatal mortality rate due to HEV per 100 000 live births	22.5 (10.5–37.5)

Abbreviation: HEV, hepatitis E virus.

^a^Projected for 2014 (Supplementary Appendix 1).

^b^The expected number of nonmaternal HEV-associated deaths for a specific age group in the hospital catchment areas was calculated by multiplying the number of nonmaternal jaundice-associated deaths in the hospital catchment areas by the proportion of HEV-associated cases among nonmaternal jaundice-associated deaths in the surveillance hospitals for that age group. For instance, for the 15- to 44-year age group, there were 158 jaundice-associated nonmaternal deaths in the catchment areas, and the proportion of HEV-associated deaths among nonmaternal deaths in that age group in the surveillance hospitals was 14.6% (Table 3), yielding an expected number of HEV-associated deaths in the catchment areas of 23.07 (ie, 158 × 0.146).

In the sensitivity analysis for a 2-year recall period, the estimated annual number of HEV-associated deaths was 1114 (95% CI, 634–1584) for the population aged ≥14 years (including 186 [95% CI, 60–476] maternal deaths), 375 (95% CI, 133–904) stillbirths, and 780 (95% CI, 337–1367) neonatal deaths (Supplementary Appendix 2). For the 1-year recall period, the estimated number of HEV-associated annual deaths was 1101 (95% CI, 422–1901) for the population aged ≥14 years (including 177 [95% CI, 43–548] maternal deaths), 412 (95% CI, 131–1092) stillbirths, and 927 (95% CI, 359–1802) neonatal deaths.

## DISCUSSION

This study provides the first measured estimate of HEV-associated mortality in Bangladesh by combining data from (1) a mortality survey covering a population >2.3 million and (2) surveillance of hospital-based jaundice in 6 hospitals across the country. In the absence of any reported HEV outbreak in Bangladesh during 2013 to 2015, we estimated that HEV infection accounted for 1766 (95% CI, 964–2635) deaths annually, including pregnant women and neonates. Additionally, we estimated 279 stillbirths per year, which are excluded from GBD estimates [10], despite their significant physical and psychological toll. Preventing HEV infections during pregnancy could prevent an estimated 1222 maternal and neonatal deaths and stillbirths annually. This population-based estimate of HEV mortality in Bangladesh can inform the cost-effectiveness of HEV vaccination and other control measures to help prioritize public health interventions.

The GBD studies estimate disease burdens, including hepatitis E, by employing models based on available data from vital registration, surveillance, and verbal autopsy [10]. The 2019 GBD study estimated 1932 HEV-associated deaths globally [10], which appears to be a significant underestimate as we identified approximately this many deaths in Bangladesh alone. The HEV mortality estimate by GBD studies appears to be sharp reduction, from 70 000 in 2005 to 50 000 in 2013, 26 100 in 2016, and 14 700 in 2017 [6–9]. This sudden decrease is likely due to methodological changes in calculation and the use of different data sources across various GBD studies. For instance, GBD studies transitioned from a single cause-of-death ensemble model (CODEm) to a 2-model hybrid approach in 2013, incorporating a global CODEm and a CODEm limited to data-rich countries [6, 7, 10]. Moreover, the 2019 GBD study considered location- and year-specific factors for virus-specific case fatality rates [10].

The 2013 GBD study estimated 38 738 viral hepatitis deaths (25 deaths/100 000 population) in Bangladesh, with 12.7% attributed to HEV, totaling 4920 HEV-associated deaths [7]. However, GBD studies conducted in 2017 and 2019 reported significantly lower figures of 227 and 111 HEV-associated deaths, respectively [9, 10]. These variations and model uncertainties have left decision makers hesitant on efficient vaccine implementation.

We note a number of study limitations. First, in the hospital catchment areas, jaundice symptoms among deceased individuals were not verified by medical professionals; instead, caregivers reported symptoms for deaths occurring within 3 years of the survey. Although our trained field team explained jaundice symptoms and laypersons can identify eye yellowing when pronounced, misclassification may have occurred. This limitation is common in mortality surveys using the verbal autopsy method. Second, promptly conducting interviews with caregivers is crucial for accurate cause-of-death identification, as delays may introduce recall bias in reported symptoms [32]. Our sensitivity analysis showed a slightly lower number of jaundice-associated deaths in the third year preceding the survey as compared with the first 2 years. While uncertain if this reflects a true change in burden or random fluctuation, we provided separate HEV mortality estimates based on 1- and 2-year recall periods, illustrating how estimates vary when restricting recall bias (Supplementary Appendix 2). Third, while the Wantai IgM anti-HEV enzyme-linked immunosorbent assay is widely accepted as being highly sensitive for diagnosing acute HEV infections, Huang et al observed that about 3% of acute viral hepatitis cases were negative for IgM anti-HEV but had a 4-fold rise in IgG anti-HEV in convalescent sera, indicating potential underestimation in our prevalence estimates due to the test's sensitivity [33]. Fourth, our study did not account for HEV-associated miscarriages, which are significant among pregnant women with HEV infection [34]. Fifth, extrapolating findings from the hospital catchment areas to the entire population of Bangladesh may have led to a potential overestimation of HEV incidence. However, the use of a “probability proportional to size sampling” approach ensured that high- and low-prevalence areas were represented, reducing bias in the overall estimates. Additionally, the rural to urban population ratio in the catchment areas closely matched the national ratio, supporting the representativeness of our estimates. Finally, this study presents the HEV burden in Bangladesh based on data 6 to 9 years old, which may not precisely reflect the current situation. Nonetheless, there are no other measured HEV mortality estimates in Bangladesh, so our estimates remain useful. Considering that there have been no targeted strategies to reduce HEV transmission since this study, it is unlikely that there has been a significant change in HEV burden in Bangladesh over this period.

This study provides a dependable estimate of HEV-associated mortality in Bangladesh, addressing the lack of reliable data on HEV-associated mortality nationally and globally [14]. Our findings highlight the substantial underestimates of HEV deaths globally, based on the latest GBD estimates published in 2020 [10], suggesting that these estimates should not be used for global policy decisions. Our data from Bangladesh can help determine the cost-effectiveness of vaccination against HEV and other interventions, including improvement of drinking water quality, for national policy makers, and our methods can be used by other countries requiring accurate HEV-associated mortality estimates.

## Supplementary Material

jiaf134_Supplementary_Data

## Supplementary Data

Supplementary materials are available at *The Journal of Infectious Diseases* online (http://jid.oxfordjournals.org/). Supplementary materials consist of data provided by the author that are published to benefit the reader. The posted materials are not copyedited. The contents of all supplementary data are the sole responsibility of the authors. Questions or messages regarding errors should be addressed to the author.

## Notes

***Financial support.*** This work was supported by the US Centers for Disease Control and Prevention.
