# Supplementary material for: Population-Based Estimates of Hepatitis E Virus–Associated Mortality in Bangladesh
Source: J Infect Dis. 2025 Mar 13;231(6):e1129–37. doi: 10.1093/infdis/jiaf134 (PMC12247814; doi:10.1093/infdis/jiaf134)
Supplement: jiaf134_Supplementary_Data [file jiaf134_supplementary_data.docx]

## Appendices

**Appendix 1: Projected population and live-births in 2014 in the catchment areas of the six acute jaundice surveillance hospitals, Bangladesh**

| **Surveillance hospitals** | **No. of unions** | **Census population^1^** | **Population projection^2^** | | | **Projected population ≥ 14 years^4^** | **Projected live-birth^5^** |
| --- | --- | --- | --- | --- | --- | --- | --- |
|  |  |  | **2012** | **2013** | **2014** |  |  |
| Bogra |  |  |  |  |  |  |  |
| Rural | 12 | 427,635 | 433,494 | 439,432 | 445,453 |  |  |
| Urban^3^ | 2 | 34,414 | - |  | - |  |  |
| ***Total*** | ***14*** |  |  |  | ***479,867*** | ***324,870*** | ***10,653*** |
| Barisal |  |  |  |  |  |  |  |
| Rural | 11 | 214,544 | 217,483 | 220,463 | 223,483 |  |  |
| Urban^3^ | 4 | 49,278 | - |  | - |  |  |
| ***Total*** | ***15*** |  |  |  | ***272,761*** | ***184,659*** | ***6,055*** |
| Kishoregonj |  |  |  |  |  |  |  |
| Rural | 6 | 153,425 | 155,527 | 157,658 | 159,818 |  |  |
| Urban^3^ | 1 | 3,645 | - |  | - |  |  |
| ***Total*** | ***7*** |  |  |  | ***163,463*** | ***110,664*** | ***3,629*** |
| Chittagong |  |  |  |  |  |  |  |
| Rural | 19 | 548,491 | 556,005 | 563,623 | 571,344 |  |  |
| Urban^3^ | 6 | 101,065 | - |  | - |  |  |
| ***Total*** | ***25*** |  |  |  | ***672,409*** | ***455,221*** | ***14,927*** |
| Sylhet |  |  |  |  |  |  |  |
| Rural | 15 | 393,194 | 398,581 | 404,041 | 409,577 |  |  |
| Urban^3^ | 4 | 47,585 | - |  | - |  |  |
| ***Total*** | ***19*** |  |  |  | ***457,162*** | ***309,498*** | ***10,149*** |
| Mitford, Dhaka |  |  |  |  |  |  |  |
| Rural | 3 | 107,617 | 109,091 | 110,586 | 112,101 |  |  |
| Urban^3^ | 8 | 199,249 | - |  | - |  |  |
| ***Total*** | ***11*** |  |  |  | ***311,350*** | ***210,784*** | ***6,912*** |
| All |  |  |  |  |  |  |  |
| Rural | 66 | 1,844,906 | 1,870,181 | 1,895,803 | 1,921,775 | *1301,042* | *42,663* |
| Urban^3^ | 25 | 435,236 | - |  | 435,236 | *294,655* | *9,662* |
| ***Total*** | ***91*** | ***2,280,142*** |  |  | ***2,357,011*** | ***1,595,697*** | ***52,326*** |
| ^1^ Population census in Bangladesh was conducted in 2011 | | | | | | | |
| ^2^ Projected population considering 1.37% growth rate | | | | | | | |
| ^3^ Population in urban areas was counted through house-to-house visits in the mortality survey | | | | | | | |
| ^4^ Projected considering 67.7% of population are ≥ 14 years | | | | | | | |
| ^5^ Live-births are projected considering crude birth rate as 22.2/1000 population | | | | | | | |

#

# Appendix 2: Estimation of HEV associated mortality in the catchment areas of six acute jaundice surveillance hospitals by recall period, 2014 (Survey period: November 2014–February 2017

| **Mortality estimation (indicators)** | **Recall period** | | | | | | |  |
| --- | --- | --- | --- | --- | --- | --- | --- | --- |
|  | **One year** |  | **Two years** |  | | **Three years** | |  |
| ***Estimated population and live births in the hospital* catchment areas**  (projected for 2014; Appendix 1) |  |  |  |  | |  | |  |
| Population aged ≥ 14 years *[P]* | 1,595,697 |  | 1,595,697 |  | | 1,595,697 | |  |
| Live births *[*$B$*]* | 52,326 |  | 52,326 |  | | 52,326 | |  |
| ***Surveillance hospitals*** |  |  |  |  | |  | |  |
| Maternal deaths |  |  |  |  | |  | |  |
| Maternal deaths with acute jaundice *[*$J_{m}$*]* | 27 |  | 27 |  | | 27 | |  |
| HEV cases among maternal deaths with acute jaundice *[*$E_{m}$*]* (95% CI) | 8 (4-13) |  | 8 (4-13) |  | | 8 (4-13) | |  |
| Non-maternal deaths |  |  |  |  | |  | |  |
| Non-maternal deaths with acute jaundice *[∑*$J_{i}$*]* | 275 |  | 275 |  | | 275 | |  |
| HEV cases among non-maternal deaths with acute jaundice *[∑*$E_{i}$*]* (95% CI) | 20 (13-30) |  | 20 (13-30) |  | | 20 (13-30) | |  |
| Stillbirths |  |  |  |  | |  | |  |
| Stillbirths delivered by mothers with acute jaundice *[*$S_{j}$*]* | 27 |  | 27 |  | | 27 | |  |
| Stillbirths delivered by HEV positive mothers *[*$S_{e}$*]* (95% CI) | 6 (3-11) |  | 6 (3-11) |  | | 6 (3-11) | |  |
| Neonatal deaths |  |  |  |  | |  | |  |
| Neonatal deaths born to mothers with acute jaundice *[*$N_{j}$*]* | 15 |  | 15 |  | | 15 | |  |
| Neonatal death cases born to HEV positive mothers *[*$N_{e}$*]* (95% CI) | 10 (6-13) |  | 10 (6-13) |  | | 10 (6-13) | |  |
| ***Hospital catchment areas*** |  |  |  |  | |  | |  |
| Maternal deaths with acute jaundice *[*$D_{m}$*]* (95% CI) | 9 (4-17) |  | 19 (11-30) |  | | 25 (16-37) | |  |
| Non-maternal deaths with acute jaundice *[∑*$D_{i}$*]* (95% CI) | 214 (186-245) |  | 437 (401-480 | |  | | 587 (401-772) | |
| Stillbirths delivered by mothers with acute jaundice *[*$S$*]* (95% CI) | 28 (19-40) |  | 51 (38-67) |  | | 57 (43-74) | |  |
| Neonatal deaths born to mothers with acute jaundice *[*$N$*]* (95% CI) | 21 (13-32) |  | 35 (24-49) |  | | 53 (40-69) | |  |
| **HEV associated mortality** |  |  |  |  | |  | |  |
| HEV associated mortality per 100,000 population aged ≥ 14 years (95% CI) | 1.0 (0.4-1.8) |  | 1.1 (0.6-1.5) |  | | 0.93 (0.6-1.3) | |  |
| Maternal mortality ratio due to HEV per 100,000 live-births (95% CI) | 5.1 (1.2-15.8) |  | 5.3 (1.7-13.7) |  | | 4.7 (1.6-11.4) | |  |
| Stillbirth rate due to HEV per 100,000 live-births (95% CI) | 11.9 (3.8-31.5) |  | 10.8 (3.8-26.1) |  | | 8.1 (2.9-19.2) | |  |
| Neonatal mortality rate due to HEV per 100,000 live-births (95% CI) | 26.7 (10.3-52.0) |  | 22.3 (9.7-39.4) |  | | 22.5 (10.5-37.5) | |  |
| **Estimated HEV associated annual deaths in Bangladesh** |  |  |  |  | |  | |  |
| Deaths among population aged ≥ 14 years | 1102 (422-1901) |  | 1114 (634-1584) |  | | 986 (599-1338) | |  |
| Maternal deaths | 177 (43-548) |  | 186 (60-476) |  | | 163 (57-395) | |  |
| Stillbirths | 412 (131-1092) |  | 375 (133-904) |  | | 279 (101-664) | |  |
| Neonatal deaths | 927 (359-1802) |  | 780 (337-1367) |  | | 780 (365-1298) | |  |
